# Tea bud pose estimation and grading detection network based on improved YOLOv7

**DOI:** 10.3389/fpls.2026.1786144

**Published:** 2026-03-12

**Authors:** Yuchen Yao, Zhiyong Gui, Haoyang Liu, Zidong Yang, Lijian Yao, Kai Li, Zhenchuan Lin, Yihu Mao, Zhijun Jia, Yang Li, Rong Ma

**Affiliations:** 1 College of Optical, Mechanical and Electrical Engineering,Zhejiang Agriculture and Forestry University, Hangzhou, China; 2 Tea Research Institute, Chinese Academy of Agricultural Sciences, Hangzhou, China; 3 National Key Laboratory for Tea Plant Germplasm Innovation and Resource Utilization, Hangzhou, China; 4 Fujian Pin Pin Xiang Tea Industry Co., Ltd., Ningde, China

**Keywords:** deep neural network, grading, lightweight, pose estimation, tea bud

## Abstract

Intelligent recognition and rapid grading of tea buds are crucial for advancing tea-picking machinery; however, complex plantation backgrounds and inconsistent bud growth have limited traditional algorithms to merely identifying picking points, neglecting bud pose and grade, which restricts harvesting efficiency. To address these challenges, we propose YOLO-PC, a deep neural network designed for simultaneous tea bud pose estimation and classification, which incorporates a dynamic snake convolution (DSConv) module for enhanced shape feature extraction, an ELASPP-CSPC attention mechanism for improved spatial pooling, and EIoU loss to accelerate regression and boost localization accuracy. Experimental results demonstrate that the model achieves detection accuracies of 91.5% for one-bud-one-leaf and 93.2% for one-bud-two-leaf scenarios, with an average keypoint detection accuracy (Pose_mAP) of 89.7% and a Normalized Mean Error (NME) of 0.047; furthermore, compared to YOLOv7-pose, it increases mean average precision by 7.26% and pose accuracy by 9.65% while reducing parameters by 14.99 M. Ablation studies confirm the superior performance of the proposed model in tea bud detection, indicating its potential to provide robust practical support for adaptive and intelligent tea harvesting systems.

## 1 Introduction

Tea and tea drinking both originated in China. In 2024, China’s total raw tea output amounted to 3.4991 million metric tons, with a year-on-year growth of 159,600 metric tons (4.78%).Due to its richness of beneficial components (e.g., catechins, cholesterenone, caffeine, inositol, folic acid, and pantothenic acid), tea is highly beneficial to human health. Tea beverages made from processed tea leaves have become one of the most popular and healthiest drinks globally (Ahammed and Li, 2022).

With ongoing economic development, the cultivation area for tea plants and the market for tea beverages have consistently expanded. Consequently, ordinary bulk teas increasingly fail to meet the consumer expectations that have shifted toward higher quality standards. This trend has led to a steadily growing demand for famous and high-quality teas (Li et al., 2022). As treasured g ems of traditional Chinese culture, these famous and high-quality teas are meticulously selected and processed to meet exacting standards. They are characterized not only by refined flavor, fresh aroma and delicate texture, but also by rich nutritional and medicinal values, offering significant health benefits (Bansal et al., 2012), such as anticancer (Jankun et al., 1997), anti-inflammatory effects (Molina et al., 2015) and antiviral effects (Yang et al., 2014), etc.

Famous and high-quality tea buds are still predominantly harvested by hand. However, the high labor costs and low efficiency of manual work cannot meet the increasing demand for famous and high-quality teas (Yan et al., 2022). Although the use of traditional tea plucking machines can improve harvesting efficiency (Yang et al., 2021), their uniform picking method without graded selection often breaks some bud-leaves and compromises the integrity of tea buds. Moreover, harvesting by cutting rather than plucking leaves can easily damage the stems and petioles, adversely affecting the sprouting of new buds (Owuor et al., 2009).Additionally, the lack of effective selection during harvest will leave some tea branches or old leaves in the harvested tea, reducing the quality of raw leaves and making them unsuitable as raw materials for high-grade renowned green teas. Therefore, achieving automation and intelligence in famous and high-quality tea picking is of great significance, and the key prerequisites for this purpose are to accurately identify tea buds and precisely locate picking points (Zhang et al., 2024; Motokura et al., 2020).

In recent years, with the rapid advancement of computer vision and deep learning technologies, research on tea leaves continues to make significant progress, vision-based automatic picking robots have garnered significant attention for recognition and harvesting of famous and high-quality teas (Liu and Wang, 2021). Gui et al. (2023) proposed a YOLOv5-based lightweight tea bud detection model, which improved recognition accuracy while reducing computational load and parameter volume. Li et al. (2023) embedded the convolutional block attention module into the path aggregation network, enhanced the feature extraction capability of the model and increased average accuracy by 1.08% compared to the original YOLOv4 algorithm. Lu et al. (2024) introduced the α-CIoU loss function to address similar background environments, and adjusted the α parameter to enhance the model ability to recognize tea buds in highly similar backgrounds. Xue et al. (2023) proposed YOLO-Tea, an improved model based on YOLOv5 that integrates ACmix, CBAM, RFB, and GCNet modules, significantly enhancing detection accuracy and efficiency. Chen and Chen (2020) proposed a two-stage deep learning approach based on Faster R-CNN and FCN, which achieves efficient detection and segmentation performance while demonstrating strong generalization across different tea cultivars.

The harvesting standards for high-grade famous and high-quality teas are particularly stringent, and typically require the selection of one-bud-one-leaf or one-bud-two-leaves while ensuring leaf integrity. However, research on graded recognition of buds and leaves remains relatively scarce. Paranavithana and Kalansuriya (2021) proposed a deep convolutional neural network (CNN)-based model to classify tea buds into suitable and unsuitable buds for picking. They employed a four-layer CNN architecture, trained it using the Adam optimizer and ReLU activation function, and achieved a final classification accuracy of 70.15%. The model also demonstrated strong generalization performance on small datasets. Xu et al. (2022) developed a detection and classification method utilizing a variable-universe-based two-level fusion network, which integrated YOLOv3 and DenseNet201. This method achieved a tea bud recognition accuracy of 95.7% and a recall rate of 94.5%.

In field environments, tea plantations present complex backgrounds and inconsistent growth patterns of tender buds. Factors such as tea variety, plant pose, size, occlusion among leaves, and variable lighting conditions contribute to relatively large localization errors for some key feature points, making the detection of tea buds and optimal picking points challenging (Chen et al., 2022; Li et al., 2023). However, traditional recognition methods focus on locating picking points while neglecting the overall posture of the bud-leaf unit. This neglect prevents the robotic end-effector from aligning with the natural growth direction of tender shoots, often resulting in partial damage or harvesting failure. To address this issue, Chen et al. (2024) proposed a tea bud keypoint detection network named TBKNet. This network reconstructs the CSPDarknet53 backbone using the concept of structural re-parameterization, and introduces a novel keypoint regression loss function that incorporates both angular and Euclidean distances to accelerate model convergence. Experimental results demonstrate that TBKNet achieves a mean average precision (mAP) of 87.1% for keypoint detection. To address the challenges of false and missed detections caused by the high visual similarity between tea buds and complex backgrounds, Liu et al. (2025) proposed TBD-Y, an automatic tea bud detection method based on YOLOv11, which significantly improves detection accuracy by integrating a Synergistic Object-Spatial Attention (SOSA) mechanism and a Global-Local Attention Guided Feature Fusion (GAGFF) strategy.

In actual picking processes, experienced tea pickers select the optimal picking method based on the natural growth pose of tea leaves, which maximizes tea quality preservation. Currently, considerable research has been conducted on pose estimation of fruits. For instance, Tianxiao Zhu et al. (2025) proposed an OBB-Pose model based on improved YOLOv8, which achieves 5-degree-of-freedom spatial pose estimation for strawberries by simultaneously detecting oriented bounding boxes and structural keypoints, combined with RGB-D point cloud information. Seung-Woo Kang et al. (2025) proposed a two-stage method for rapid pose estimation of oriental melon fruit-stem pairs, and utilized class activation maps (CAM) for weakly supervised fruit localization, reducing annotation costs while maintaining real-time performance.

However, research focusing on the growth poses of tea buds and leaves remains relatively scarce. For example, Chen et al. (2023) used a depth camera with an improved YOLOv5 to detect tea buds and estimate their 3D poses. Wang et al. (2024) applied pose estimation techniques to detect and analyze Yinghong No. 9 tea leaves and optimized the algorithm using TKS_NMS; their proposed algorithm effectively estimated the poses of tea leaves. Yifan Cheng et al. (2023) optimized the keypoint detection branch in Mask R-CNN by introducing a novel anchor generation method, increasing the keypoint localization accuracy of the model to 85.9%. Although the above studies have investigated the poses of buds and leaves, they provide only rough estimations for the one-bud-one-leaf category and lack extension to other quality grades, such as one-bud-two-leaves. Furthermore, these studies focus on the bud and leaf parts of the tea shoot, neglecting stem pose estimation. Consequently, they fail to accurately represent the natural growth pose of the entire tea shoot. Additionally, the performances of the existing models in complex environments require improvement, as target detection accuracy, keypoint precision, and robustness against interference remain suboptimal.

To address the above issues, this study proposes a tea bud detection network model named YOLO-PC (You Only Look Once- Pose estimation and Classification), which can grade and identify tea buds while estimating their poses. A dataset of ‘Longjing 43’ and ‘Zhongcha 108’ with multiple targets, poses and sizes was created, labeling targets of one-bud-one-leaf and one- bud-two-leaves and annotating keypoints for both categories. By improving YOLOv7, the model can simultaneously grade and identify buds and leaves while estimating their poses, and enhances the accuracy of bud and leaf detection and keypoint prediction.

This research lays a visual foundation for automated harvesting of tea buds in field environments, contributing to the modernization and intellectualization of tea production.

The main contributions are as follows:

1. A mixed dataset of ‘Longjing 43’ and ‘Zhongcha 108’ with multiple quantities, lighting conditions, and weather scenarios was established for pose estimation of one-bud-one-leaf and one-bud-two-leaves.
2. This study proposes a deep neural network model named YOLO-PC. The model ability to capture shape information of tea shoots was enhanced by introducing the dynamic snake convolution (DSConv) module into YOLOv7 and adding an attention mechanism between the Backbone network and the Head network.
3. The original CIoU loss calculation was replaced with EIoU calculation, which improved the model regression capability for target bounding boxes and the accuracy and stability of small target detection and grading.
4. Based on the pose estimation results of tender buds, the offset angle between tea shoots and the vertical direction was calculated, facilitating the determination of subsequent picking strategies and enabling more precise picking.

Experimental results show this method can effectively detect tea poses in images and distinguish between one-bud-one-leaf and one-bud-two-leaves. The improved tea detection model YOLO-PC demonstrates 9.65% improvement in keypoint detection performance with 18.75% reduction in model parameters. Comparative experiments indicate YOLO-PC significantly outperforms other object detection algorithms.

The second part introduces the creation of the relevant dataset and the proposed improved method for tea bud grading detection and pose estimation based on YOLOv7. The third part discusses the experimental results and visualizations. The fourth section provides a summary of the study and discusses future optimization directions.

## 2 Materials and methods

### 2.1 Data collection

**Figure 1:**
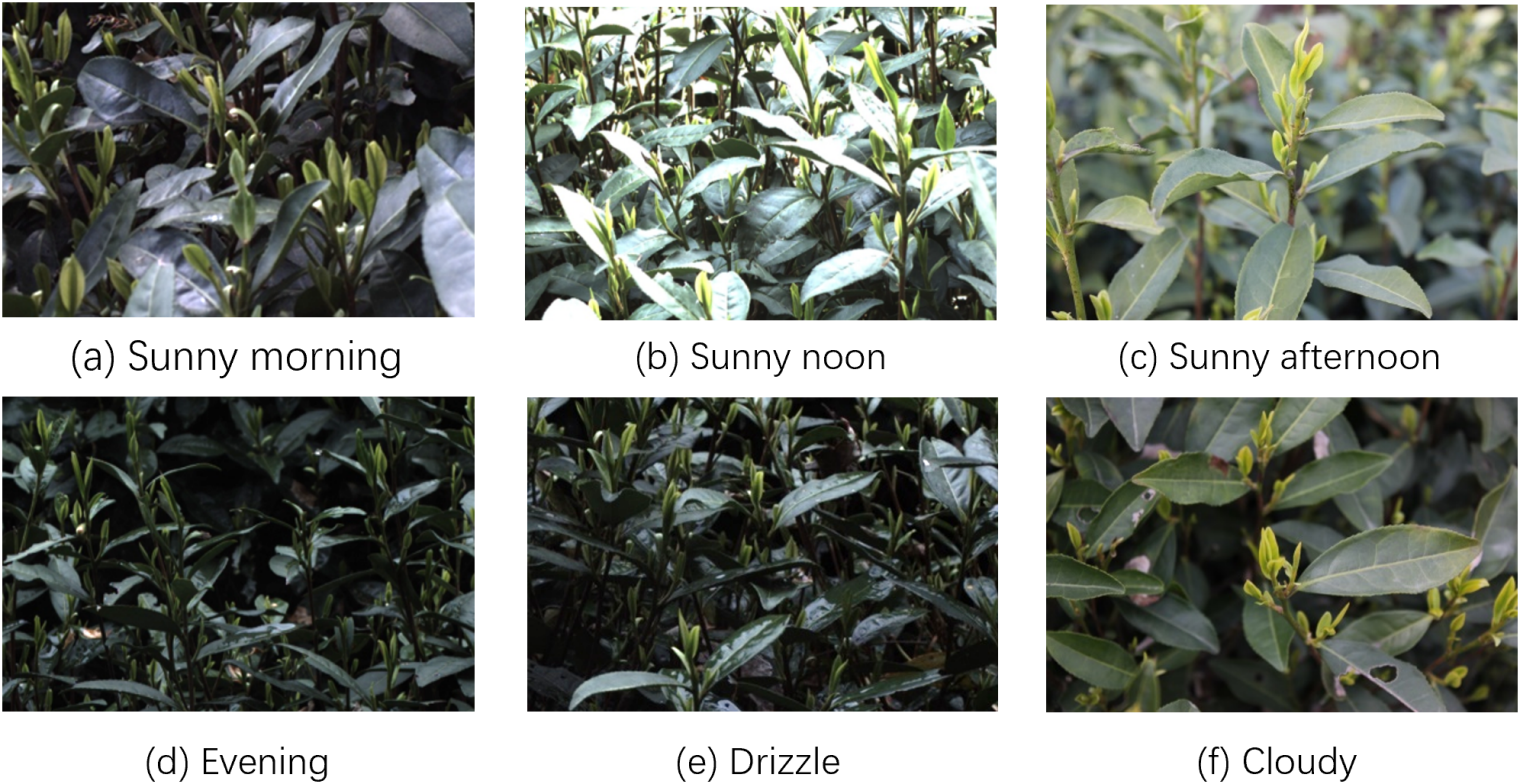
Images taken in various environments: **(a)** Sunny morning, **(b)** Sunny noon, **(c)** Sunny afternoon, **(d)** Evening, **(e)** Drizzle, **(f)** Cloudy.

From March 15, 2024 to March 25, 2024, data were collected in a tea plantation near the Tea Research Institute of the Chinese Academy of Agricultural Sciences in Xihu District, Hangzhou City, Zhejiang Province, China (120°5′33.763″ E, 30°10′48.025″ N). An industrial-grade Mech-Eye PROS 3D camera (Mech-Mind Robotics (Beijing) Co., Ltd.) was used to capture 800 images of ‘Longjing 43’ and ‘Zhongcha 108’ at a resolution of 1920×1200 from a 45° angle. The specific parameters of the camera are listed in **Table 1**. To enhance the robustness and generalization capability of the visual recognition module, factors such as the working hours of the tea-picking robot and weather conditions were carefully considered during dataset construction. On a sunny day, the light varies greatly from morning to night. The light on a sunny morning is soft (**Figure 1a**). The light is the strongest at noon on a sunny day, when the young shoots are less distinguishable from the new leaves, and the leaves are reflected and form white light (**Figure 1b**). The light in the afternoon is weaker than at noon, with some shadows (**Figure 1c**). The light intensity is generally low in the evening, and the colors of the new and old leaves are similar (**Figure 1d**). In the case of drizzle, the light is soft and uniform throughout the day, but there will be a lot of raindrops on the leaves (**Figure 1e**). It is cloudy and the light is soft and uniform throughout the day (**Figure 1f**). The images of tea shoots taken under different time periods and weather conditions show the variations in light intensity and angle significantly affect the recognition and pose estimation of tea shoots.

**Table 1:** Parameters of Mech-EyePROS industrial 3D camera.

Mech-Eye PRO S	
Recommended working distance range (mm)	500~1000
Near field of view	370×240@0.5m
Far field of view	800×450@1.0m
Resolution	1920 × 1200
Number of pixels (MP)	2.3
Z-axis single-point repeatability	0.05 mm (within 1 m distance)
VDI/VDE measurement accuracy	0.1mm @1m
Acquisition time (s)	0.3~0.6
Dimensions (mm)	265 × 57 ×100
Weight (kg)	Approx. 1.6
Light source	Blue LED (459 nm, RG2)/White LED (RG2)
Input	24VDC, 3.75A
Communication interface	Gigabit Ethernet

**Figure 2:**
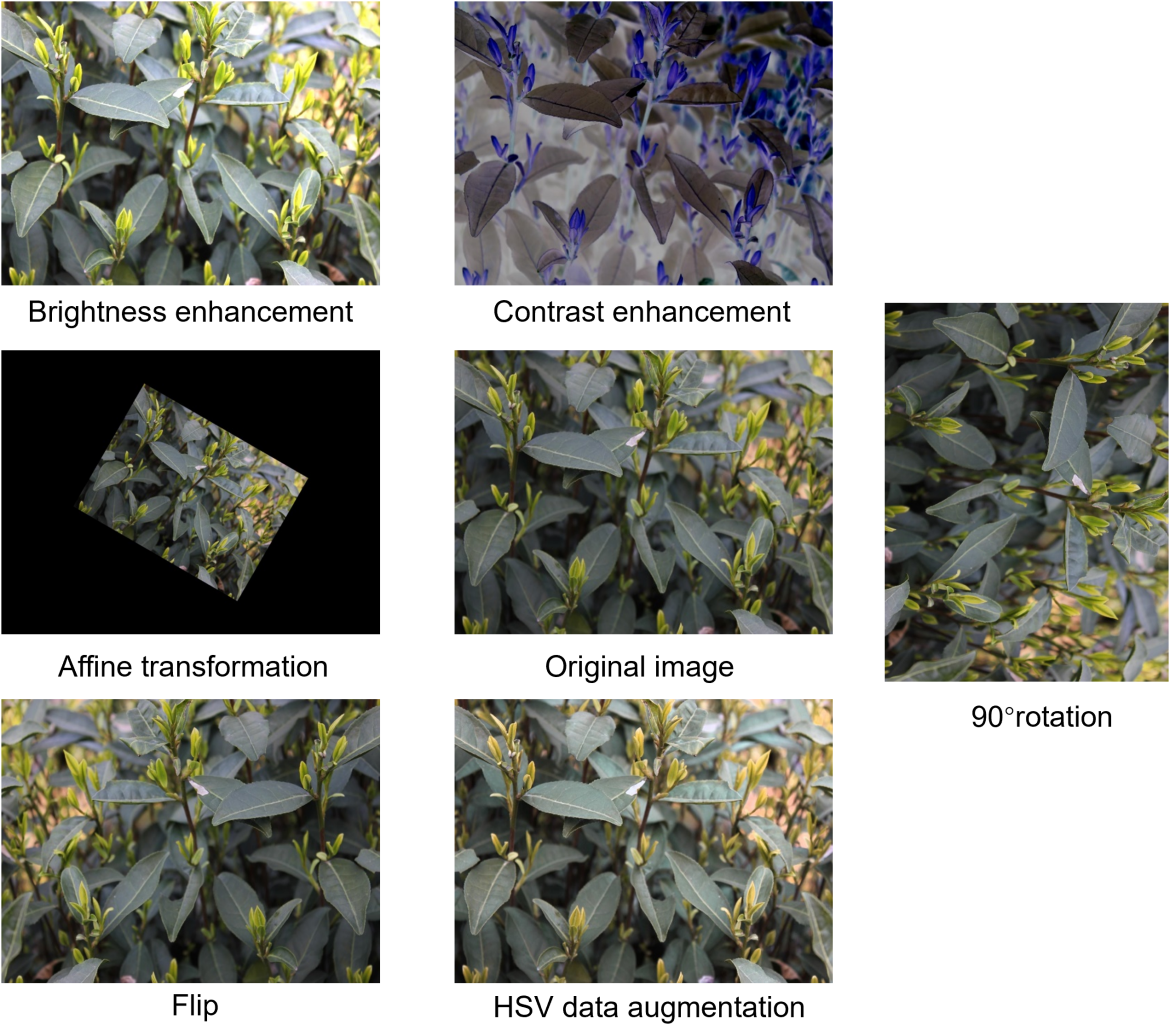
Data-enhanced images.

The diversity of data types can significantly affect model performance. A dataset containing too few samples may lead to overfitting (Shorten and Khoshgoftaar, 2019). However, obtaining more image data requires additional human and material resources. Therefore, this study expands the dataset through image augmentation methods, mainly including brightness enhancement, contrast enhancement, rotation (90°), affine transformation, HSV data augmentation, and horizontal flipping. The results after transformation are shown in **Figure 2**. The augmented dataset contains totally 2,000 images.

### 2.2 Dataset preparation

To ensure an accurate evaluation of the real-world generalization capability of the model, we adopted a rigorous data partitioning strategy to mitigate the risk of information leakage. First, 100 completely unprocessed samples were randomly selected from the entire set of 800 original images to form an independent subset. This subset was designed to simulate the real data distribution that the model would encounter in practical applications. The remaining 700 original images were further divided into training and validation subsets at a 9:1 ratio, comprising 630 and 70 samples, respectively.

Data diversity significantly impacts model performance. Insufficient data can easily lead to overfitting, while acquiring more image data implies more investments in human and material resources (Shorten and Khoshgoftaar, 2019). Therefore, we employed various data augmentation techniques, including brightness enhancement, contrast enhancement, 90° rotation, affine transformation, HSV color space enhancement, and horizontal flipping. Example results of these transformations are shown in **Figure 2**. During data preprocessing, augmentation operations were applied solely to the training subset, expanding its size from 630 to 1,600 samples, and an equal proportion of samples was generated by each augmentation method. Both the validation and test subsets were maintained in their original states without any augmentation, and potential information leakage paths that could arise from augmented variants of homologous samples distributed across different data subsets were fundamentally eliminated.

After the above processing, the final dataset comprises totally 1,770 images, including 1,600 augmented samples in the training set, 70 original samples in the validation set and 100 original samples in the test set. To standardize the input dimensions and reduce computational complexity, all images underwent spatial resolution normalization, and resized to a fixed size of 640 × 640 pixels prior to being input into the network.

**Figure 3:**
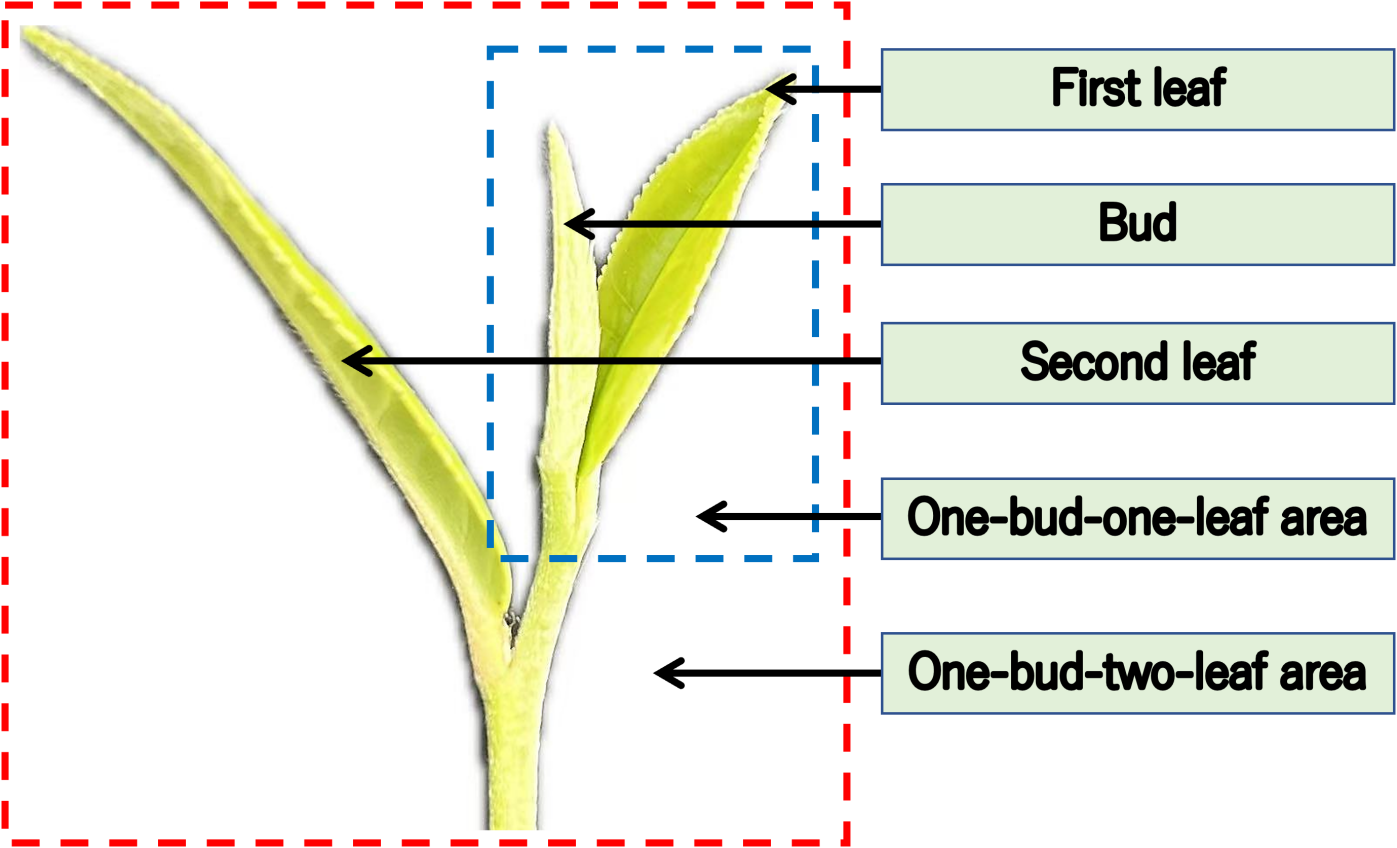
Schematic diagram of Longjing 43 shoots.

**Figure 4:**
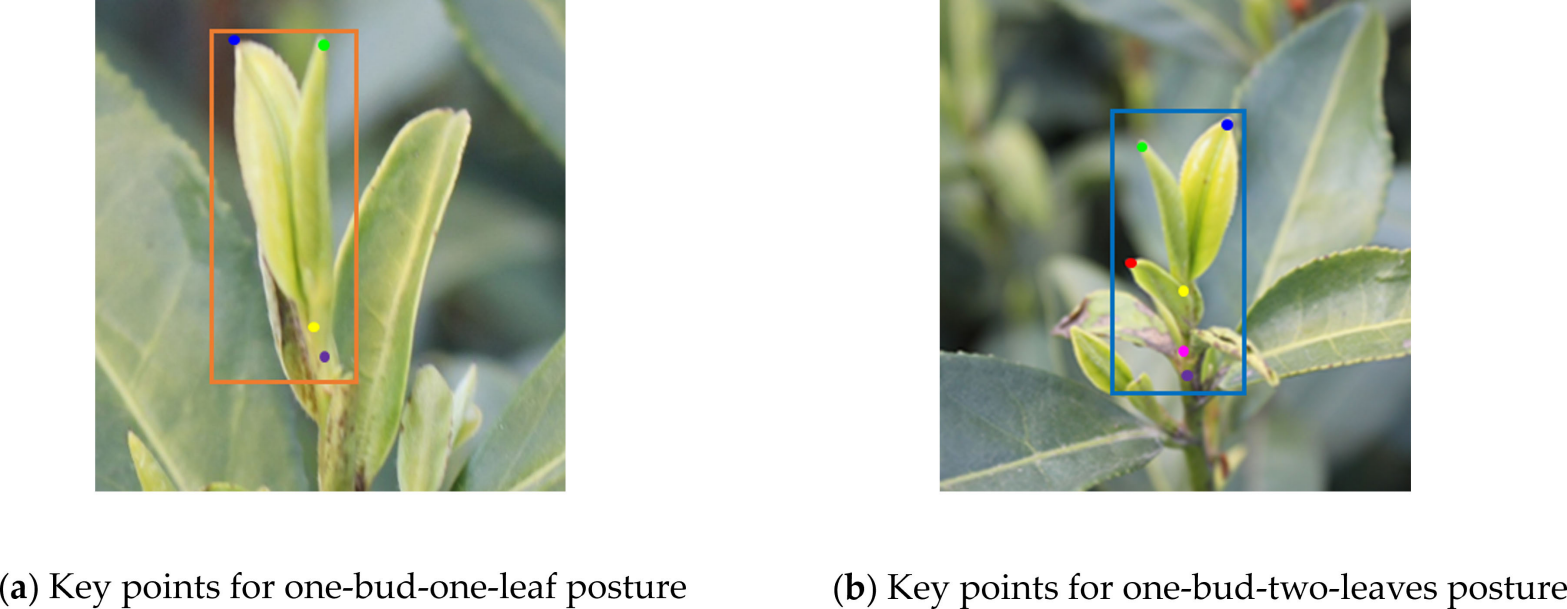
Schematic diagrams of key points for one-bud-one-leaf and one-bud-two-leaves poses. **(a)** Key points for one-bud-one-leaf pose. **(b)** Key points for one-bud-two-leaves pose.

Accurately differentiating and evaluating tea grade can ensure consistency and meet market demand for a particular tea type (Cao et al., 2024). According to the current demand of the tea market, famous and high-quality tea is mainly made of single bud, one-bud-one-leaf, and one-bud- two-leaves through different processes. To consider the different needs of tea picking, the objects identified here are one-bud-one-leaf and one-bud-two-leaves (**Figure 3**). A complex dataset was created by blending images captured from different time periods, quantities, and weather conditions, enabling tea picking robots to adapt to multiple working conditions for identification and picking. The data were labeled with the open-source application Labelme, the data format was json, one-bud-one-leaf was labeled as Bud1, and one-bud-two-leaves was labeled as Bud2. An excessive number of keypoints leads to increased model complexity and prolonged processing time, whereas insufficient keypoints fail to accurately represent the actual pose of buds and leaves. Therefore, this study selected four and six keypoints to characterize the poses of one-bud-one-leaf and one-bud-two-leaves structures, respectively. The keypoint annotations are as follows: the top of the tea bud (Green), the top of the first leaf (Dark blue), the top of the second leaf (Red), base of the first leaf (Yellow), base of the second leaf (Pink), and Picking point of the tea bud (Purple). The schematic diagrams of the key points in the tea poses of Bud1 in **Figure 4a** and of Bud2 in **Figure 4b** include 4 and 6 key points respectively. The specific parameters are shown in **Table 2**. Given the irregular growth of large-leaved tea leaves and the serious mutual occlusion, the tea leaves with more than 60% occlusion during data labeling were not labeled.

**Table 2:** Labeled parameters.

Name	Color	Type
Bud1	Orange	Rectangle
Bud2	Blue	Rectangle
Bud_top	Green	Point
First leaf_top	Dark blue	Point
Second leaf_top	Red	Point
First leaf_bottom	Yellow	Point
Second leaf_bottom	Pink	Point
Picking point	Purple	Point

### 2.3 YOLOv7-pose model improvement strategy

The YOLOv7 object detection algorithm was proposed by Wang et al. Notably, the YOLO algorithm series were proven to achieve excellent generalization performance across various datasets and object categories (Hu et al., 2018; Wang et al., 2023). YOLOv7 is the first to involve the model reparameterization technique into its network architecture, which helps enhance the expressive capability of the model without increasing computational complexity. Furthermore, YOLOv7 has the ELAN (Extended efficient Layer Aggregation Network) architecture. This architecture enables efficient detection performance and maintains low computational complexity by optimizing the network structure and computational flow.

As an end-to-end key point detection algorithm, YOLOv7-Pose adopts an innovative multi-branch stacked structure, endowing it with sufficient feature extraction capability. The YOLOv7-Pose model consists of three components: Backbone (backbone network), Neck (neck network), and Head (detection head). These network layers collectively constitute a reference feature set, which captures the semantic information of tea buds and leaves. Specifically, the Neck layer is an enhanced feature extraction network unique to YOLOv7-Pose, and fuses three effective feature layers obtained from Backbone. This fusion integrates multi-scale feature information of tea buds and leaves, effectively improving the accuracy and generalization ability of the model. The function of Head is to analyze these fused feature points and determine whether the predefined bounding boxes on these points correspond to the tea bud and leaf objects in the image, achieving the detection of tea buds and leaves.

**Figure 5:**
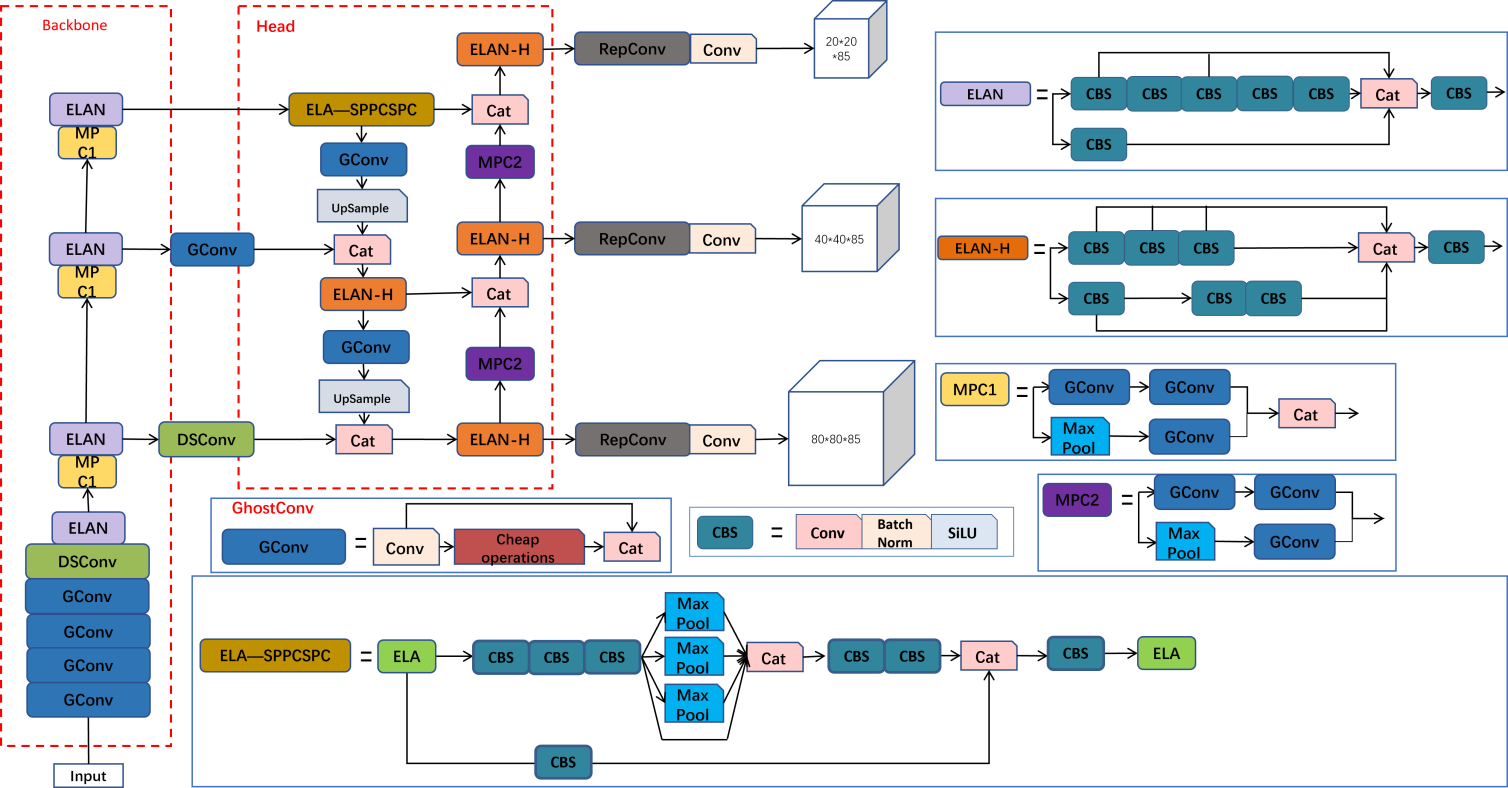
Structure of YOLO-PC network.

The original YOLO-Pose primarily targets the detection of 17 keypoints of the human body. We focus on detecting 4 keypoints for one-bud-one-leaf, and 6 keypoints for one-bud-two-leaves. Therefore, the original YOLOv7-Pose was modified to adapt to the application scenario. The proposed YOLO-PC network consists of a backbone network, an enhanced feature extraction network, and a prediction network. The backbone network is composed of a lightweight GhostNet, which reduces computational complexity. DSConv, which is more sensitive to tubular structures such as buds and leaves, is introduced to replace some standard convolutions in the backbone feature extraction network. Moreover, the complex background environment of tea plantations, significant variations in lighting intensity at different time points and under different weather conditions, and interference factors such as varying growth densities and poses of buds and leaves shall be considered. Therefore, the ELA module is integrated into SPPCSPC to assign higher weights to the information of tea buds, improving the accuracy of tea bud detection. Finally, EIoU is introduced to replace the original CIoU loss function, and thus enables faster matching between predicted and ground truth boxes and effectively enhances the convergence speed of the model. The structure of the YOLO-PC network is shown in **Figure 5**.

#### 2.3.1 A lightweight backbone network GhostNet based on deep separable convolution

**Figure 6:**
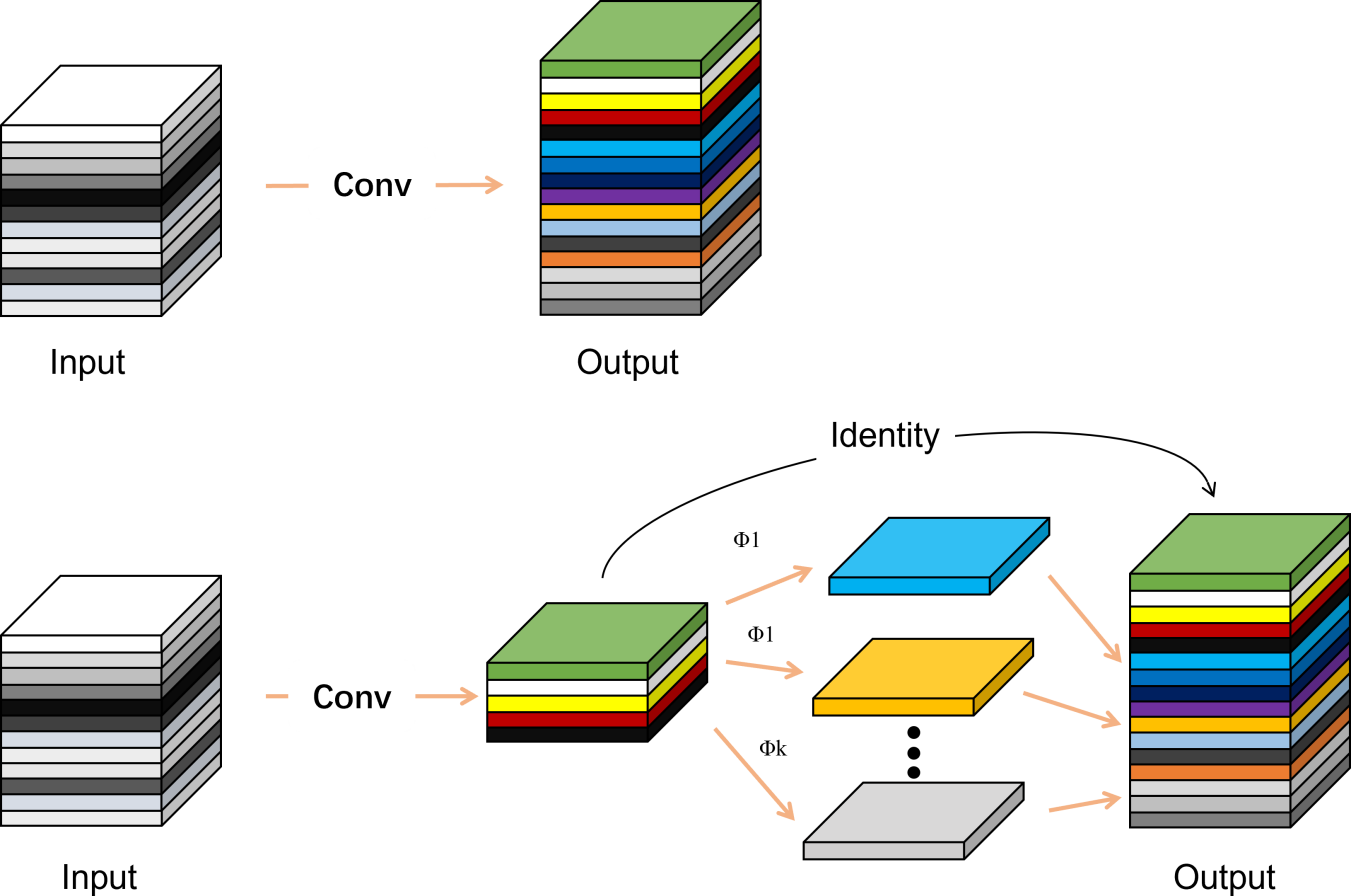
Comparison of standard convolution (top) and Ghost_conv (bottom).

To facilitate the model deployment on mobile devices and simplify the computation amount, the Ghost_conv module in GhostNet, a lightweight backbone network based on deep separable convolution, was used to replace the CBS in the YOLOv7 model Modules (Han et al., 2014). First, a small amount of ordinary convolution calculations is conducted to extract the basic features, and a feature layer with a small number of channels is generated. Then additional features are generated from these preliminary features through linear mapping, which can replace the generation of similar feature maps, so that the Ghost convolution can reduce the computation and parameters while maintaining the performance. Finally, the two sets of feature maps are stitched together to form a multi-channel feature layer, which is transmitted to the subsequent network. The ordinary convolution and Ghost_conv are compared in **Figure 6**.

Ghost_conv further reduces the computational effort through inexpensive operations, and the parameters can be further compressed. Ghost_conv is compared to generating ordinary convolutions with the same number of channels and the same width and height of feature layers and FLOP. The formula for ordinary convolutional FLOP is calculated as Equation 1:

(1) $$\begin{array}{c} Flop1=n\times h^{\prime }\times w^{\prime }\times c\times k\times k \end{array}$$

and the formula for Ghost_conv FLOP is calculated as Equation 2:

(2) $$\begin{array}{c} Flop2=\;\frac{n}{s}\times h^{\prime }\times w^{\prime }\times c\times k\times k+\left(s-1\right)\times h^{\prime }\times w^{\prime }\times \frac{n}{s}\times d\times d \end{array}$$

where *n* and *c* are the number of output and input channels, respectively, $h^{\prime }$ and $w^{\prime }$ are the height and width of the output feature, respectively, k is the size of the convolution kernel, s is the number of features generated by depthwise convolution in Ghost_conv, and d is the size of a depthwise convolution kernel.

(3) $$\begin{array}{l} \text{rs}=\frac{\text{Flop}1}{\text{Flop}2}=\frac{\text{n}\times h^{\prime }\times w^{\prime }\times c\times k\times k}{\frac{n}{s}\times h^{\prime }\times w^{\prime }\times c\times k\times k+\left(s-1\right)\times h^{\prime }\times w^{\prime }\times \frac{n}{s}\times d\times d}\\ =\frac{c\times k\times k}{\frac{1}{s}\times c\times k\times k+\frac{s-1}{s}\times d\times d}\approx \frac{s\times c}{s+c-1}\approx s\; \end{array}$$

Therefore, theoretically, the numbers of parameters and computations used in Ghost convolution are about the same as those of $\frac{1}{s}$ traditional convolution.

#### 2.3.2 DSConv

The target of tea shoots is small, irregular, and the growth poses are quite different. Traditional convolution operation is affected by different detection tasks owing to its fixed receptive field, and can hardly deal with small targets. Especially slender targets, there are certain limitations. In conventional convolution, a standard 3×3 2D convolutional kernel K is calculated as Equation 4:

(4) $$\begin{array}{c} K=\left\{\left(x-1,y-1\right),\left(x-1,y\right),\ldots \left(x+1,y+1\right)\right\} \end{array}$$

where x is the abscissa of the pixel (horizontal direction of the image) and y is its ordinate (vertical direction of the image). Hence, on a 2D image, the center of the convolution kernel is located in pixel (x,y), and the coordinates of the adjacent 8 pixels are considered at the same time.

**Figure 7:**
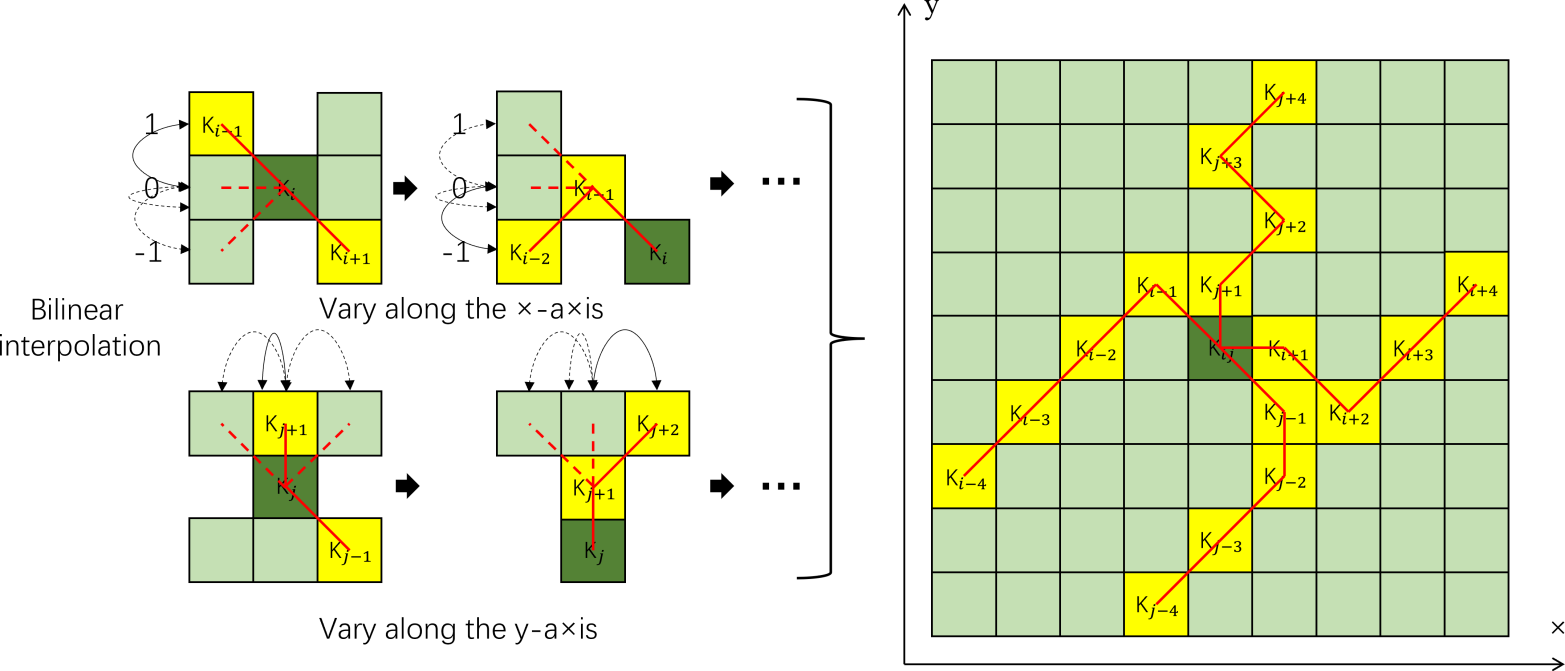
Schematic diagrams of coordinate calculation for DSConv kernel and optional receptive fields.

DSConv is a CNN with dynamic and deformable kernels. By incorporating geometric topology constraints and a tree-structured guidance mechanism, this architecture adaptively focuses on slender curved local features while leveraging the unique properties of tubular structures in feature extraction, feature fusion, and loss constraints. To balance the deformation flexibility of convolutional kernels with structural stability, DSConv employs a progressive iterative optimization strategy. Specifically, it sequentially localizes adjacent key regions and constrains the kernel offset range, maintaining spatial coherence during feature learning and preventing feature response dispersion due to excessive deformation. The model iteratively processes each target point while observing the next position (**Figure 7**). This mechanism ensures the flexible adaptation of the kernel along tubular structures while preventing perceptual focus deviation from the target through path continuity supervision (Qi et al., 2023).

where ${\text{K}}_{\text{i}}\pm \text{c}=\left({\text{x}}_{\text{i}}\pm \text{c},{\text{y}}_{\text{i}}\pm \text{c}\right)$ is the position of each grid in K, where c = {0,1,2,3,4}indicates the horizontal distance from the center grid. The dashed lines represent possible change positions, while the solid lines indicate definite change positions.

With a convolutional kernel in size 9 as an example, the $K_{i}\pm c$ selection of each grid position in convolution kernel K is a cumulative process. K starts from the center grid, and the previous grid position is used as a reference to form the position of the next off-center grid. The position $\;K_{i}+1$ is determined by an increment of an offset Δ={δ|δ∈[−1,1]} relative to $K_{i}$. Therefore, the offsets need to be accumulated to ensure that the convolution kernel conforms to the linear morphological structure. In the diagram above, the change in the x-axis is calculated as Equation 5:

(5) $$K_{i\pm c}\left\{ \begin{array}{l} (x_{i+c},y_{i+c})=\left(x_{i}+c,y_{i}+\sum_{i}^{i+c}\Delta y\right)\\ \left(x_{i-c},y_{i-c}\right)=\left(x_{i}-c,y_{i}+\sum_{i-c}^{i}\Delta y\right) \end{array}\right.$$

The change in the y-axis is calculated as Equation 6:

(6) $$K_{j\pm c}\left\{ \begin{array}{l} (x_{j+c},y_{j+c})=\left(x_{j}+\sum_{j}^{j+c}\Delta x,y_{j}+c\right)\\ \left(x_{j-c},y_{j-c}\right)=\left(x_{j}-\sum_{j-c}^{j}\Delta x,y_{j}+c\right) \end{array}\right.$$

where ${\text{ x}}_{\text{i}+\text{c}}$ and ${\text{y}}_{\text{i}+\text{c}}$ are the horizontal and vertical coordinates, respectively, of the grid position ${\text{K}}_{\text{i}\pm \text{c}}$ when varying along the x-axis; ${\text{ x}}_{\text{i}+\text{c}}$ and ${\text{y}}_{\text{i}+\text{c}}$ are the horizontal and vertical coordinates, respectively, of the grid position ${\text{K}}_{\text{j}\pm \text{c}}$ when varying along the y-axis; Δy is the y-axis offset when varying along the x-axis, and Δx is the x-axis offset when varying along the y-axis.

Precisely because DSConv is more free than standard convolution but not too free like deformable convolution, this method ensures not only the degree-of-freedom of adaptation to the structure but also the continuity of feature perception, making it easier to learn slender structural features such as tea buds.

#### 2.3.3 Characteristic pyramid of convergence ELA attention mechanism

**Figure 8:**
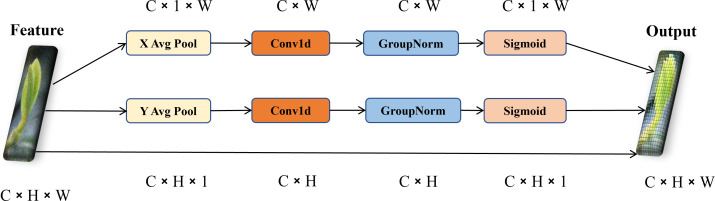
Structural diagram of ELA.

In real tea garden environments, the background is highly complex, and the growth of tea buds and leaves is irregular with diverse shapes. These factors significantly challenge bud and leaf recognition and pose estimation. Therefore, the efficient local attention (ELA) module (Xu et al., 2025) is introduced to enhance the model responsiveness to local features while minimizing the increase in computational load. The ELA module focuses on a more localized range compared to traditional self-attention mechanisms. It can precisely localize regions of interest by effectively encoding two one-dimensional positional feature maps and maintaining the channel dimensions of the input feature map while retaining lightweight characteristics. The ELA module is similar to the coordinate attention (CA) and introduces a strip-shaped pooling operation in spatial feature processing. This operation extracts direction-sensitive features along the horizontal and vertical axes. The method employs elongated convolutional kernels to establish long-range spatial correlations, and effectively suppresses interference from irrelevant regions in feature representations, enhancing the discriminative power of target localization features. During feature fusion, this module processes directional feature vectors separately and achieves cross-directional feature interaction through multiplicative operations, ultimately precisely focusing on the coordinate information of key regions. The structure of ELA is shown in **Figure 8**.

The SPPCSPC block, a special convolutional layer in YOLOv7, is used to extract image features and achieve object detection. SPPCSPC blocks are aligned with spatial pyramid pooling (SPP) and cross stage partial connection (CSPC). Firstly, the input feature map is divided into blocks, and then different sizes in each block are pooled. Next, the pooled results are spliced according to the positions of the original blocks, and finally convolution operation is carried out. These steps make it possible to extract richer feature representations and improve the performance of object detection without changing the size of the feature map.

**Figure 9:**
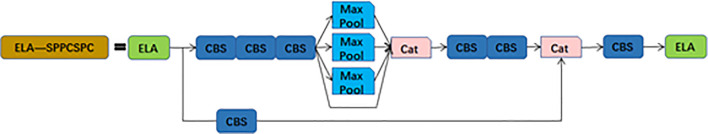
Structure diagram of ELA-SPPCSPC module.

Since the effectiveness of SPPCSPC feature extraction slightly depends on the quality of the input features, SPPCSPC may not significantly improve the results, if the feature extraction before SPPCSPC performs poorly. To effectively address the limitations of the original convolutional layers in the network in extracting inter-channel and spatial feature information, the ELA module is integrated into SPPCSPC to enhance feature extraction. Additionally, the ELA module is added again after the last CBS module to optimize multi-scale information fusion and improve model robustness. Finally, the ELA-SPPCSPC is proposed, and its structure diagram is shown in **Figure 9**.

#### 2.3.4 Improved optimization of loss function

The loss function measures the degree of agreement between the model and the data, and can guide model improvement during training. YOLOv7 adopts the CIoU loss function, an improved loss calculation method (Zheng et al., 2020). The CIoU loss function introduces the distance between the centers and the aspect ratio, and further considers shape and pose, better optimizing the bounding box coordinates and improving localization accuracy. The formula for CIoU is calculated as Equations 7–9:

(7) $$\begin{array}{c} {\text{Loss}}_{obj}=1-IoU+\frac{\rho^{2}\left(b,b^{gt}\right)}{c^{2}}+\alpha \gamma \end{array}$$

(8) $$\begin{array}{c} \gamma =\frac{4}{\pi^{2}}{\left(\tan^{-1}\frac{w^{gt}}{h^{gt}}-\tan^{-1}\frac{w}{h}\right)}^{2} \end{array}$$

(9) $$\begin{array}{c} \alpha =\frac{\gamma }{\left(1-IoU\right)+\gamma } \end{array}$$

**Figure 10:**
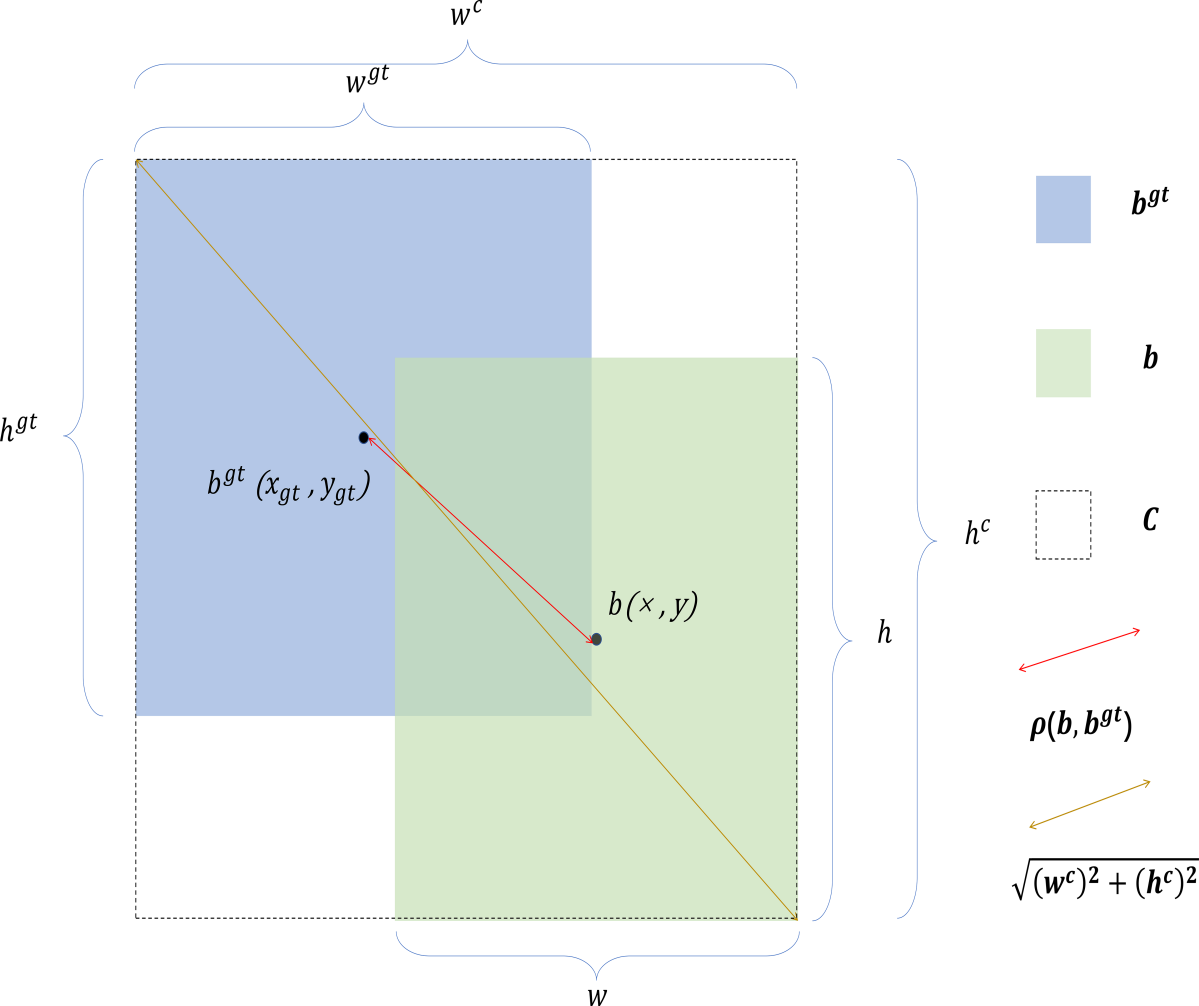
Structure diagram of EIoU loss.

Although CIoU introduces aspect ratio matching, it overlooks the individual differences in width and height. To address this issue, Zhang et al. (2022) proposed the efficient intersection over union (EIOU) loss function. EIoU incorporates a penalty term C based on the aspect ratio, which allows the shape differences of bounding boxes to be better reflected. EIoU more emphasizes the shape alignment between predicted and ground truth boxes, where a smaller area ratio results in a larger penalty during scoring, encouraging the model to generate more accurately shaped predicted boxes. To further enhance the model detection accuracy without increasing network complexity, we introduce EIoU Loss. EIoU consists of three components: IoU loss, distance loss, and width-height loss. The addition of width-height loss directly minimizes the differences in height and width between the predicted bounding box and the ground truth bounding box, leading to faster convergence and better localization results. The structure diagram of the EIoU loss is shown in **Figure 10**. The formulas for EIoU are is calculated as Equations 10 and 11:

(10) $$\begin{array}{c} L_{EIOU}=L_{IOU}+L_{dis}+L_{asp} \end{array}$$

(11) $$\begin{array}{c} L_{EIOU}=1-IoU+\frac{\rho^{2}\left(b,b^{gt}\right)}{{\left(w^{c}\right)}^{2}+{\left(h^{c}\right)}^{2}}+\frac{\rho^{2}\left(w,w^{gt}\right)}{{\left(w^{c}\right)}^{2}}+\frac{\rho^{2}\left(h,h^{gt}\right)}{{\left(h^{c}\right)}^{2}} \end{array}$$

where $\text{ρ}\left(\text{b},{\text{b}}^{gt}\right)$ is the Euclidean distance between the center points of b and ${\text{b}}^{\text{gt}}$; ${\text{w}}^{\text{c}}$and ${\text{h}}^{\text{c}}$are the width and height of the smallest enclosing box C that covers ${\text{b}}^{\text{gt}}$ and b, respectively; $\text{ ρ}\left(\text{w},{\text{w}}^{\text{gt}}\right)$ and $\text{ρ}\left(\text{h},{\text{h}}^{\text{gt}}\right)$ are the differences in width and height between the predicted box and the ground truth box.

### 2.4 Evaluation indices

Precision, Recall, AP@0.5and mAP@0.5 were employed as evaluation metrics for detection of tea buds and their key points, and to assess the accuracy, omission rate, and overall performance of tea bud and key point detection, these evaluation metrics are calculated according to Equations 12–15:

(12) $$\begin{array}{c} Precision=\frac{\text{TP}}{\text{TP}+\text{FP}} \end{array}$$

(13) $$\begin{array}{c} Recall=\frac{\text{TP}}{\text{TP}+\text{FN}} \end{array}$$

(14) $$\begin{array}{c} AP=\int_{0}^{1}P\left(R\right)dR\times 100\% \end{array}$$

(15) $$\begin{array}{c} mAP@0.5=\frac{\sum_{1}^{N}AP}{N}=\;\frac{\sum_{1}^{N}\int_{0}^{1}P\left(R\right)dR}{N} \end{array}$$

True Positive Region: Positive sample is predicted to be positive samples.

False Positive Region: Negative samples are predicted to be positive samples.

False Negative region: Positive samples are predicted to be negative samples.

True Negative Region: Negative samples are predicted to be negative samples.

APK (average precision of key points) is defined as the average distance of all predicted key points from the real key points, its expression is given by Equation 16:

(16) $$\begin{array}{c} APK=e^{-\frac{{\left(kpx^{p}-kpx^{t}\right)}^{2}+{\left(kpy^{p}-kpy^{t}\right)}^{2}}{s^{t}}} \end{array}$$

where ${\text{kpx}}^{\text{p}},{\text{kpy}}^{\text{p}}$ are the coordinates of the prediction key points; ${\text{ kpx}}^{\text{t}},{\text{kpy}}^{\text{t}}$ are the coordinates of the true key points; ${\text{s}}^{\text{t}}$ is the area of the true bounding box. This term is introduced to account for the amplification of the same error effect on small targets. As ${\text{s}}^{\text{t}}$ increases, the overall influence decreases. Conversely, as ${\text{s}}^{\text{t}}$ decreases, the impact of distance becomes more pronounced.

The Normalized Mean Error (NME) is a metric used to quantify the discrepancy between predicted and ground-truth keypoint coordinates. A lower NME value indicates higher localization accuracy, reflecting reduced average error in tea bud keypoint prediction. The specific calculation is presented in Equation 17.

(17) $$NME=\frac{1}{N}\sum_{i=1}^{N}\frac{\|kp_{i}^{p}-kp_{i}^{t}{\|}_{2}}{d}$$

where N is the total number of keypoints per object; $kp_{i}^{p}=\left(kpx_{i}^{p},kpy_{i}^{p}\right)$denotes the coordinates of the i-th predicted keypoint. $kp_{i}^{t}=\left(kpx_{i}^{t},kpy_{i}^{t}\right)$denotes the coordinates of the i-th ground-truth keypoint. d is the normalization factor.

## 3 Results and discussion

### 3.1 Experimental setup

All experiments in this section were based on a Pytorch deep learning framework, the programming language was Python, and the compilation languages were Python 3.9, PyTorch 1.110, and CUDA 11.3. The learning rate was set to 0.01, the training period was 300 epochs, and the batch size was 16. The main conFigureuration of the engineering machine used here was: 13th Gen Intel(R) Core(TM) i9-13900K 3.00 GHz CPU, and 64.0 GB RAM. The operating system was Win11, and the GPU was NVIDIA GeForce RTX4060 64G.

### 3.2 Ablation test results

Ablation studies involve adding or removing specific features from a detection algorithm to evaluate their impact on the algorithm’s performance (Lawal et al., 2021).To verify whether each improvement contributes to the performance enhancement compared to the original YOLOv7, we conducted ablation experiments on the constructed dataset by sequentially adding the corresponding modules to the original YOLOv7 for training and testing. In YOLO-PC, detection boxes and keypoints are simultaneously output through parallel prediction heads with shared features in a single forward pass. To comprehensively and objectively evaluate multi-task performance, we employ multi-dimensional metrics for quantitative analysis: Box_mAP@0.5 measures the average precision of detection boxes; Pose_mAP@0.5 assesses keypoint localization accuracy; Normalized Mean Error (NME) calculates the normalized positioning deviation of keypoint coordinates; APK further validates the overall accuracy of keypoint detection; and GFLOPs evaluates model complexity.

As shown in **Table 3**, after the original convolution was replaced with Ghost_conv, accuracy slightly sacrificed compared to the baseline YOLOv7 model, but the parameter count was reduced by 30.64 M, and FLOPs were reduced by 40.9 G. After the ELA mechanism was integrated into the SPPCSPC feature pyramid, Box_mAP and Pose_mAP increased by 4.35% and 5.1%, respectively, with a minor increase in parameters and FLOPs. Although the incorporation of DSConv introduced additional computational load compared to the original model, Box_mAP and Pose_mAP were improved by 6.32% and 8.6% respectively. The introduction of the ELA mechanism and DSConv led to an increase in model complexity, but significantly enhanced detection accuracy. When EIoU was adopted as the loss function, Box_mAP and Pose_mAP both showed a slight improvement without increasing model complexity, while all other parameters remained unchanged. Based on the ablation test, all proposed improvements in this study contribute to the overall performance enhancement of the model compared to the original baseline.

**Table 3:** Ablation test results.

Tag	Basic model	+ Ghost	+ ELA	+ DSCconv	+ EIoU	AP@0.5		Box_mAP@0.5	Params (M)	FLOPs (G)	APK	Pose_mAP@0.5
						Bud1	Bud2					
1	✓					82.7	89.5	86.1	79.93	101	0.817	79.6
2	✓	✓				79.5	86.6	83.05	49.29	60.1	0.801	78.4
3	✓	✓	✓			88.8	90.9	89.85	50.34	60.5	0.884	82.4
4	✓	✓	✓	✓		90.9	92.2	91.55	64.94	115.2	0.907	89.5
5	✓	✓	✓	✓	✓	91.5	93.2	92.35	64.94	115.2	0.909	89.7

To validate the comprehensive performance of the YOLO-PC model, we compared it with six detection models (**Table 4**): Keypoint RCNN, YOLOv5s6-Pose, YOLOv7-w6-pose, YOLOv8l-pose, YOLOv12n-pose,and YOLO-PC. The Box_mAP of the YOLO-PC model is 30.53%, 22.23%, 4.57%, 7.26%,3.53% and 2.7% higher than those of Keypoint RCNN, YOLOv5, YOLOv7, YOLOv8 and YOLOv12, respectively. The GFLOP of YOLO-PC is lower than those of Keypoint RCNN and YOLOv8, but is relatively close to those of YOLOv5,YOLOv7 and YOLOv12. Although the YOLO-PC model exhibits a slight increase in parameter count compared to YOLOv12, it achieves substantial parameter reductions of 59.17%, 15.33%, 18.75%, and 6.42% relative to Keypoint R-CNN, YOLOv5, YOLOv7, and YOLOv8, respectively. YOLO-PC exhibits performance similar to YOLOv8x-pose and YOLOv12n-pose in terms of APK, while showing significant improvement compared to Keypoint RCNN, YOLOv5s6-Pose, and YOLOv7-w6-pose. Additionally, YOLO-PC achieves a higher Pose_mAP@0.5 than other models. With an NME of 0.047, YOLO-PC demonstrates at least a 7.8% reduction in error compared to other models. Although the detection speed of the model is 30.3 frames per second, which is lower than that of some competing models, it remains sufficient to meet the real-time detection requirements for tea plants. Therefore, considering mAP, GFLOPs, Params, FPS and NME together, YOLO-PC achieves the best overall performance.

**Table 4:** Comparison of experimental results among different models.

Model	Params (M)	FLOPs (G)	AP@0.5		Speed (FPS)	APK	Pose_mAP@0.5	NME
			Bud1	Bud2				
Keypoint RCNN	159.05	384.86	70.4	71.1	12	80.1	71.3	0.074
YOLOv5s6-Pose	76.7	111.4	72.7	78.4	45	79.4	77.1	0.068
YOLOv7-w6-pose	79.93	101	82.7	89.5	41.6	81.7	79.6	0.064
YOLOv8x-pose	69.4	126	88	90.4	60.7	88.6	82.4	0.067
YOLOv12n-pose	42.3	81.3	88.4	90.9	48.6	89.7	88.3	0.051
YOLO-PC	64.94	115.2	91.5	93.2	30.3	90.9	89.7	0.047

### 3.3 Visualization and analysis

**Figure 11:**
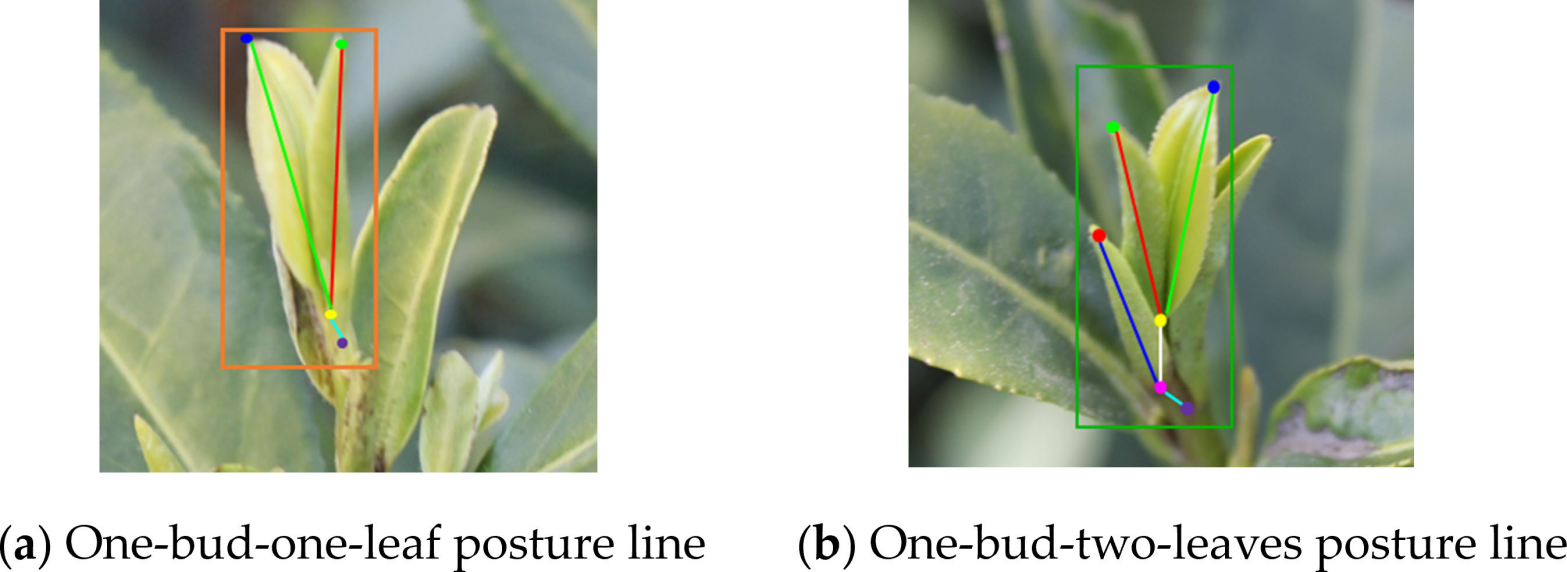
Visualization schematic of bud and leaf pose lines. **(a)** One-bud-one-leaf pose line. **(b)** One-bud-two-leaves pose line.

We further analyzed the visualization of tea leaf pose estimation. Although deep learning models can achieve high accuracy in detection tasks, the interpretability of their network processing remains insufficient. Therefore, we also analyzed the visualization of tea leaf object detection to enhance model interpretability. The parameters of the pose lines used for tea pose estimation are shown in **Table 5**, and the visualization is displayed in **Figure 11**.

**Table 5:** Visualization of bud and leaf pose lines.

Category of buds and leaves Line color	Bud1	Bud2
Red	(Bud_top, First leaf_bottom)	(Bud_top, First leaf_bottom)
Green	(First leaf_top, First leaf_bottom)	(First leaf_top, First leaf_bottom)
Blue		(Second leaf_top, Second leaf_bottom)
White		(First leaf_bottom, Second leaf_bottom)
Cyan	(First leaf_bottom,Picking point)	(Second leaf_bottom, Picking point)

**Figure 12:**
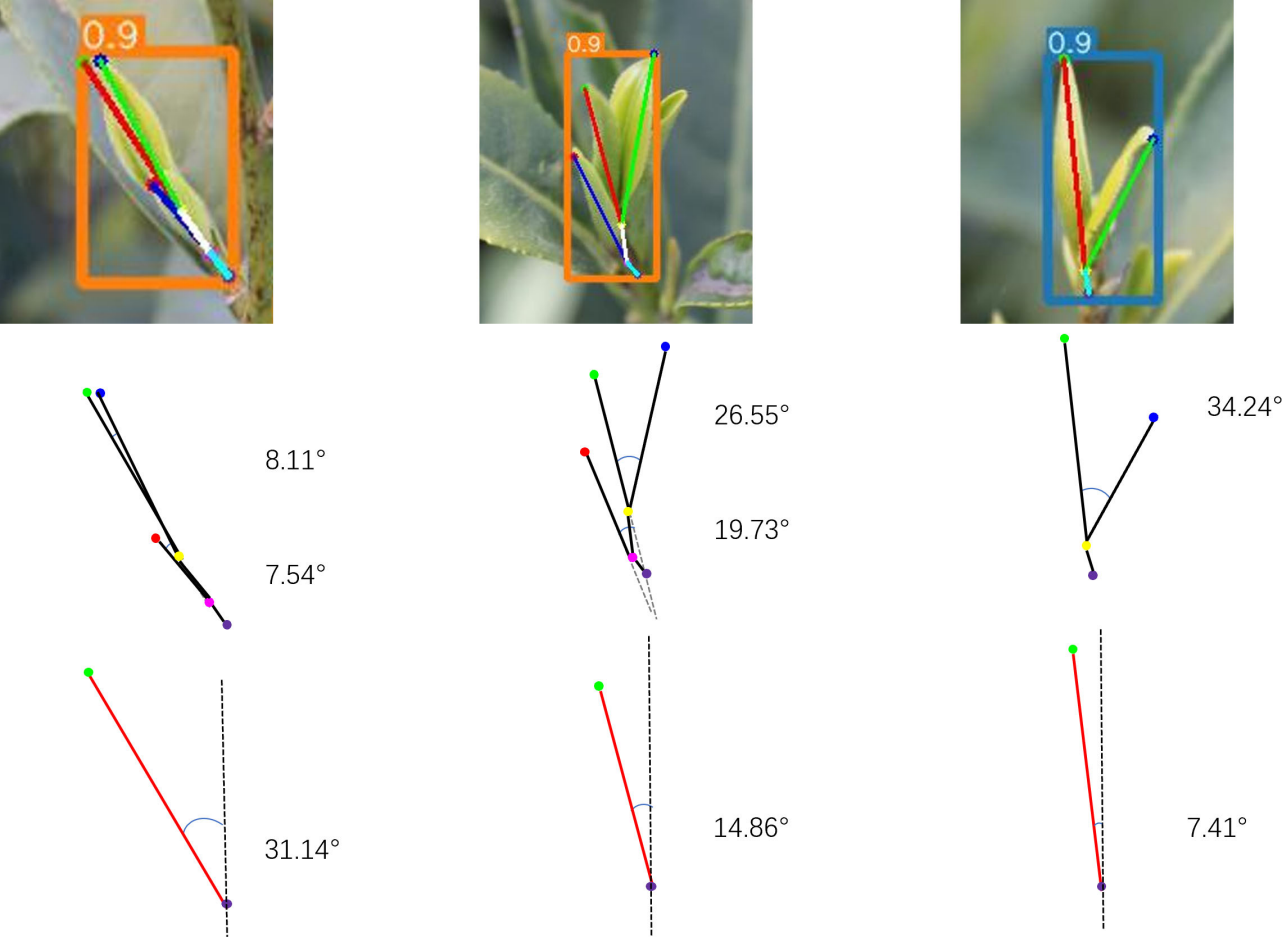
Diagram of bud and leaf pose angle estimation.

By detecting key points and lines, we can analyze the poses of tea leaves, including angle, length, and structure. Moreover, the ripeness of tea leaves was further determined by analyzing the lengths of tea buds and side leaves, and by analyzing the angle between tea buds and side leaves (Lai et al., 2022). The spreading condition of the leaves was obtained by measuring the angles between the pose line of the bud and the pose lines of the first and second leaves (**Figure 12**). Since the line connecting the bud tip and the picking point can roughly represent the overall pose of the bud and leaves, this line was used as the overall pose line to simplify calculations. The angle between this pose line and the vertical reference line was measured to determine the approximate deviation of the bud and leaves. The pose points on the image can be combined with 3D point cloud data to obtain the true three-dimensional pose of tea buds, providing a data foundation for subsequent picking strategies.

**Figure 13:**
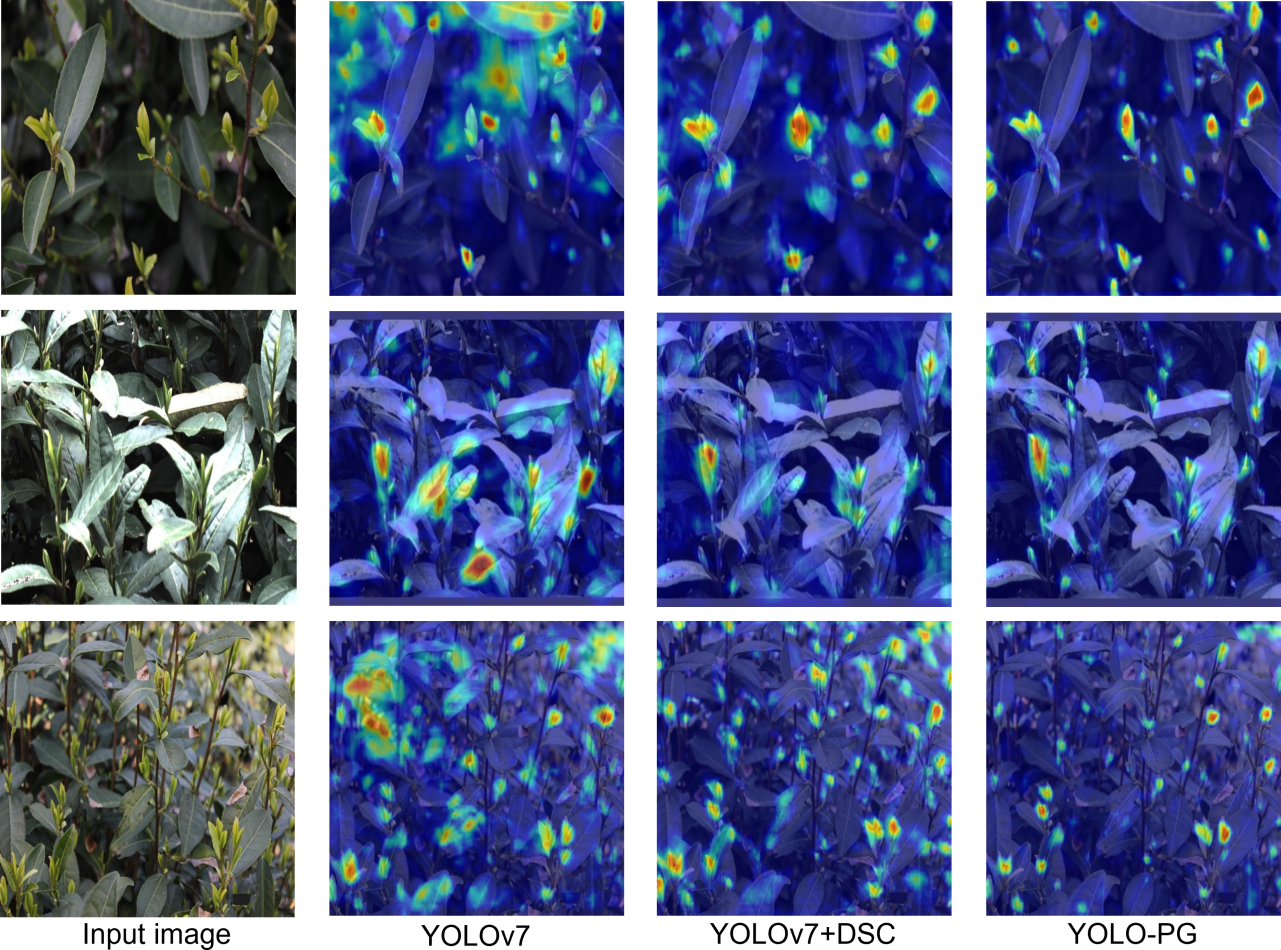
Heatmaps under different improvements.

Visualizing feature maps can help researchers understand the feature extraction capability of the backbone network. To better comprehend the learning ability of the proposed model for detecting tea buds and their picking points, Gradient-weighted CAM (Grad-CAM) was used to visualize the detection results of the YOLOv7, YOLOv7+DSC, and YOLO-PC models (Selvaraju et al., 2017). Grad-CAM flows the gradients of the target concept into the final convolutional layer of the model, generating localization maps displayed in the form of weights (**Figure 13**). Here, smaller weights correspond to bluer regions in the attention map, while larger weights correspond to redder regions, indicating these regions are more significant in the detection decision-making process for tea buds and their keypoints (Zhao et al., 2023).

Different bud-leaf density scenarios were visualized. The heatmaps show the original YOLOv7 model focuses on an overly broad area, lacking concentration (**Figure 13**). After incorporating snake-shaped dynamic convolution into the model, the model reduces attention to background images and focuses more on the elongated tea buds. Finally, the heatmap of the proposed model shows the model focus on irrelevant information is further reduced after integrating ELA into SPPCSPC and adopting EIoU as the loss function. Evidently, its attention on buds, leaves, and their key points becomes more concentrated and accurate. The heatmap visualizations additionally verify that the feature extraction capability of the proposed model for bud-leaf targets and keypoint detection is generally stronger than that of the unmodified YOLOv7.

**Figure 14:**
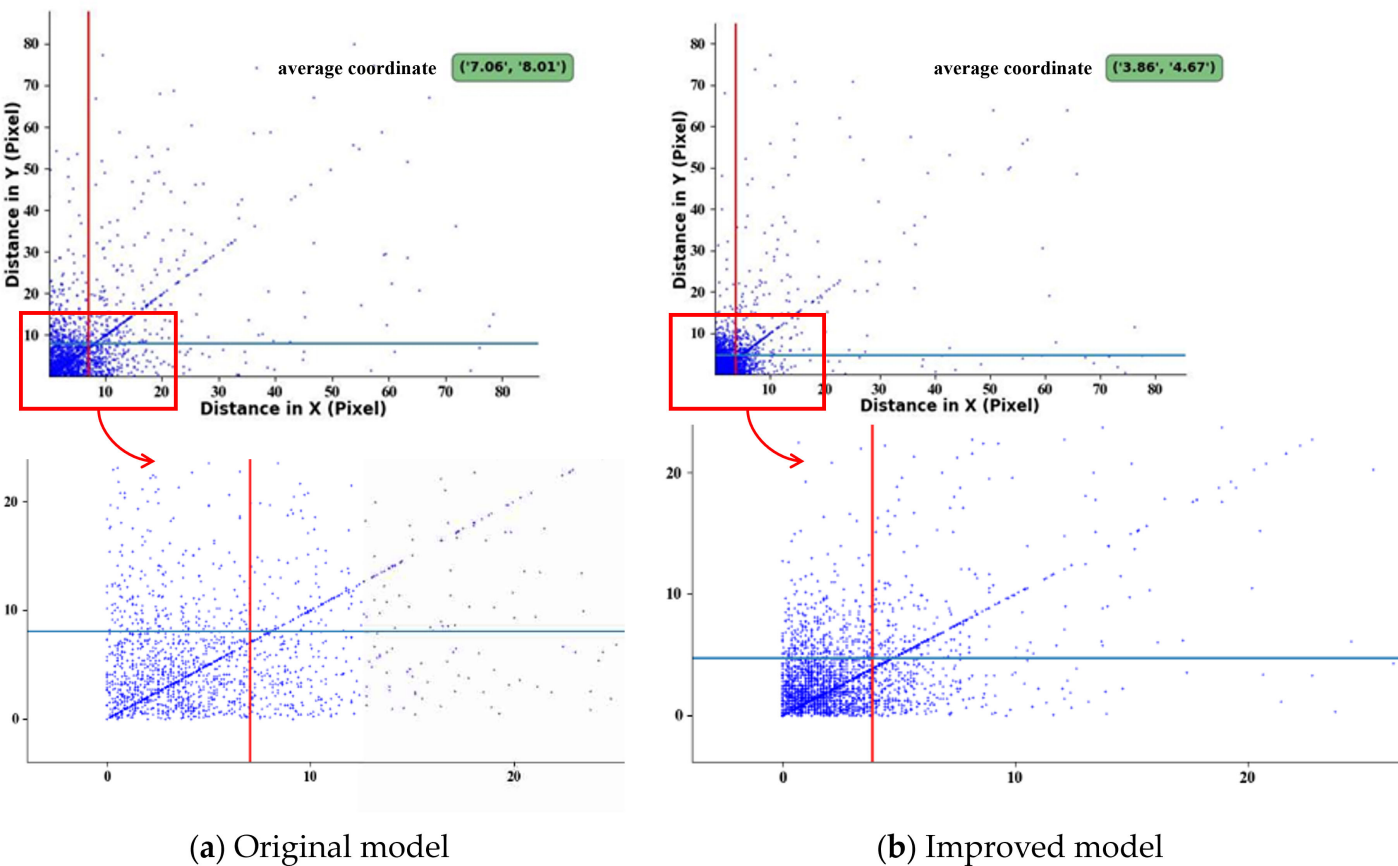
Distribution diagram of Euclidean distances between predicted coordinates and ground-truth coordinates of key points. **(a)** Original model. **(b)** Improved model.

To clearly demonstrate the accuracy of key point prediction by the YOLO-PG model (**Figure 14**), the Euclidean distance between the predicted coordinates and the ground-truth coordinates of each key point, referred to as deviation points, was calculated and plotted in the pixel coordinate system. The mean coordinates of these deviation points were displayed in the green-annotated region at the upper-right corner, with their central tendency indicated by the intersection point of the red vertical and blue horizontal reference lines.

The deviation points of the original YOLOv7 model are relatively far from the origin and are dispersed (**Figure 14a**). In contrast, the deviation points of the YOLO-PC model are mostly concentrated near the origin (**Figure 14b**). This comparison indicates the YOLO-PC model significantly improves keypoint prediction compared to the original model, and exhibits greater robustness against interference.

### 3.4 Verification of model performance under different lighting conditions

**Figure 15:**
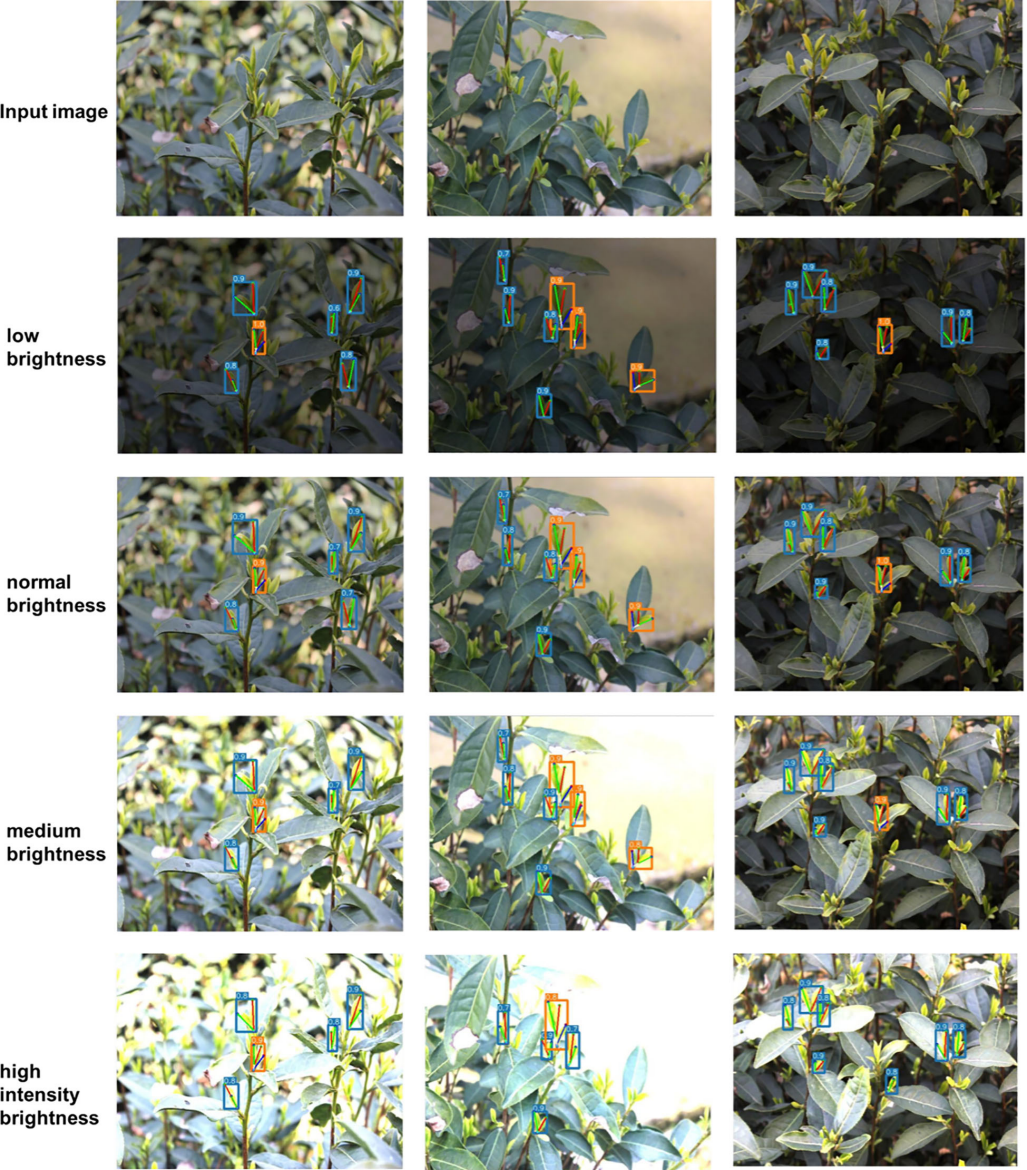
Detection results under different lighting conditions.

Since lighting conditions can significantly impact recognition, the detection performance under various lighting conditions (low, medium, and high brightness) was evaluated to understand the effect of lighting intensity on model accuracy. First, the images were converted to grayscale, and then all pixels in the grayscale images were traversed to calculate their average values. The grayscale value of each image was used as the standard for lighting intensity. Although the grayscale value ranges from 0 to 255, excessively low or high brightness is not meaningful for reference. Therefore, the intensity was divided into low brightness (20-44), normal brightness (45-121), medium brightness (122-167), and high intensity brightness (168-196). Then the proposed model was used to detect each image, and the recognition results are shown in **Figure 15**. The detection accuracy of the model was not significantly affected under low or medium brightness conditions, indicating strong robustness of the proposed model. However, some instances of missed detection and false detection still occurred under high-intensity lighting.

### 3.5 Performance evaluation of the model under varying occlusion levels

In real tea garden environments, tea buds and young leaves are often subject to varying degrees of occlusion due to factors such as interwoven branches and leaves, dense growth, or camera angles. To comprehensively evaluate the robustness and reliability of the proposed YOLO-PC model in complex scenarios, this section presents a specialized validation experiment designed for different occlusion levels, focusing on the “one bud and one leaf” and “one bud and two leaves” structures.

**Figure 16:**
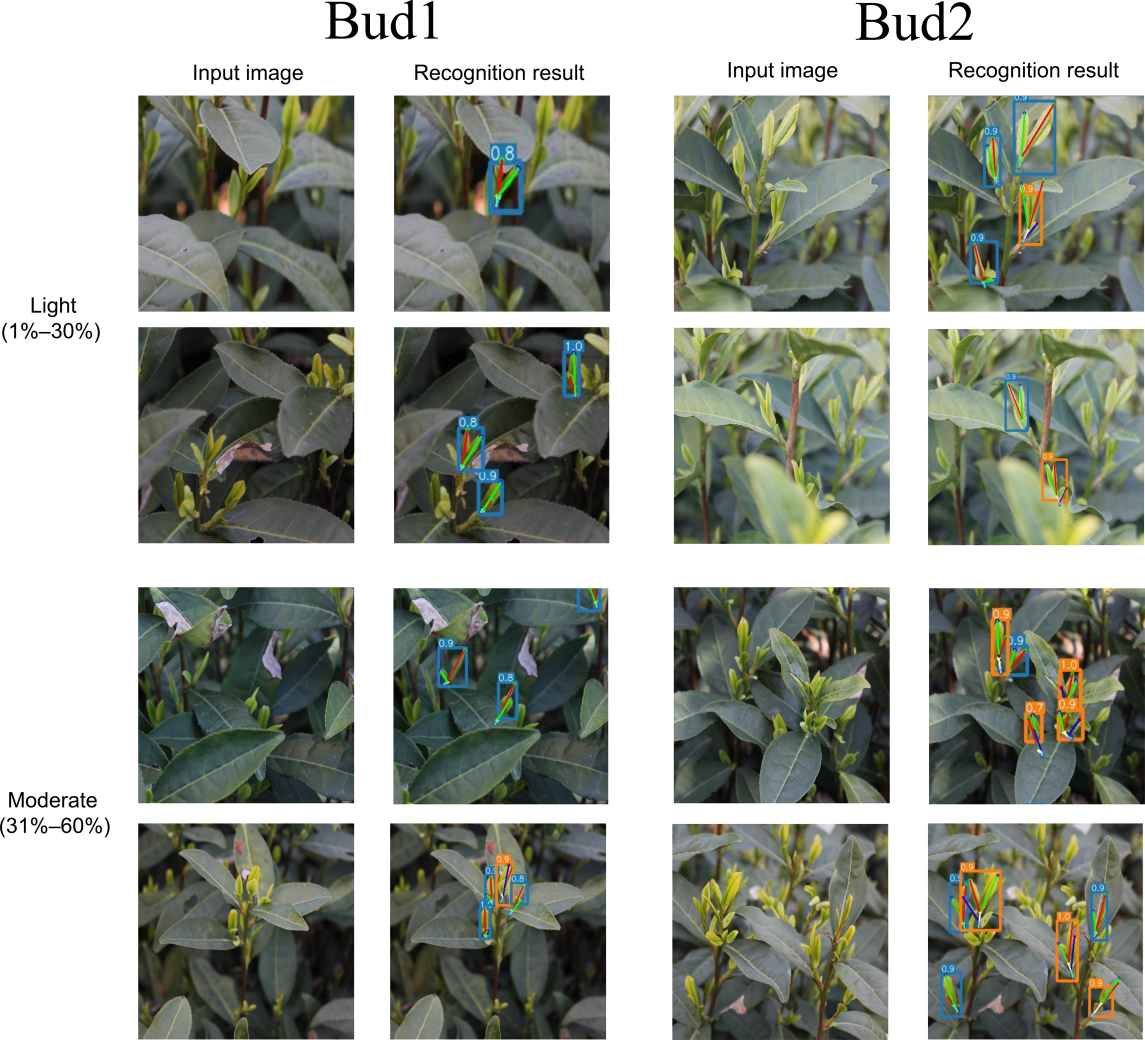
Typical prediction results for “one bud and one leaf” and “one bud and two leaves” structures under moderate and severe occlusion conditions.

Given that only a portion of the buds in each image are occluded to varying degrees while most buds remain visible, this experiment specifically analyzes the occluded buds within the images. The samples in the test set are categorized into three groups based on the target occlusion level: no occlusion (0%), light occlusion (1%–30%), and moderate occlusion (31%–60%). **Figure 16** illustrates typical prediction results for the two structures under moderate and severe occlusion conditions.

**Table 6** presents the performance of YOLO-PC on tea bud instances under varying occlusion levels.

**Table 6:** Performance of YOLO-PC on tea bud instances under varying occlusion levels.

Occlusion level	Bud type	Instances	AP@0.5	APK
No occlusion (0%)	Bud1	413	92.5	92.1
	Bud2	324	94.2	91.3
Light (1%–30%)	Bud1	381	90.7	89.5
	Bud2	304	90.6	85.7
Moderate (31%–60%)	Bud1	237	84.6	85.2
	Bud2	158	83.2	81.3

Based on the experimental results presented in **Table 6**, the performance of the YOLO-PC model for the “one bud and one leaf” and “one bud and two leaves” tea shoot structures under varying occlusion levels is analyzed as follows.

Under occlusion-free conditions, the model achieves optimal performance. For “one bud and one leaf,” the AP@0.5 reaches 92.5% and APK attains 92.1%. For “one bud and two leaves,” AP@0.5 is 94.2% and APK is 91.3%, indicating that the model exhibits exceptionally high accuracy in detection and pose estimation under ideal visibility conditions. As occlusion increases, all evaluation metrics show a declining trend, yet the decrease remains controlled, demonstrating robust performance. Under light occlusion (1%–30%), the APK for “one bud and one leaf” decreases by approximately 3.4 percentage points to 89.5%, while for “one bud and two leaves,” due to its more complex structure, APK declines to 85.7%—still maintaining a relatively high level. This suggests that light occlusion has limited impact on the model. In moderate occlusion scenarios (31%–60%), performance further decreases, but YOLO-PC retains usable accuracy: the APK for “one bud and one leaf” is 85.2%, and for “one bud and two leaves” it is 81.3%. Notably, the decline in keypoint recognition accuracy for “one bud and two leaves” is more pronounced than for “one bud and one leaf.” This reflects that the former, with a greater number of keypoints and more complex spatial topology, is more susceptible to localization deviations when partial structures are occluded.

In summary, across a continuous range from no occlusion to moderate occlusion, YOLO-PC consistently maintains stable and high performance in both detection and keypoint localization for the two tea shoot structures. Particularly in practical agricultural applications, where most occlusion falls within the light to moderate range, the model’s performance is sufficient to support high-accuracy shoot identification and picking-point localization tasks. This validates its reliability and practicality in real-world tea garden environments.

### 3.6 Failure case visualization and analysis

**Figure 17:**
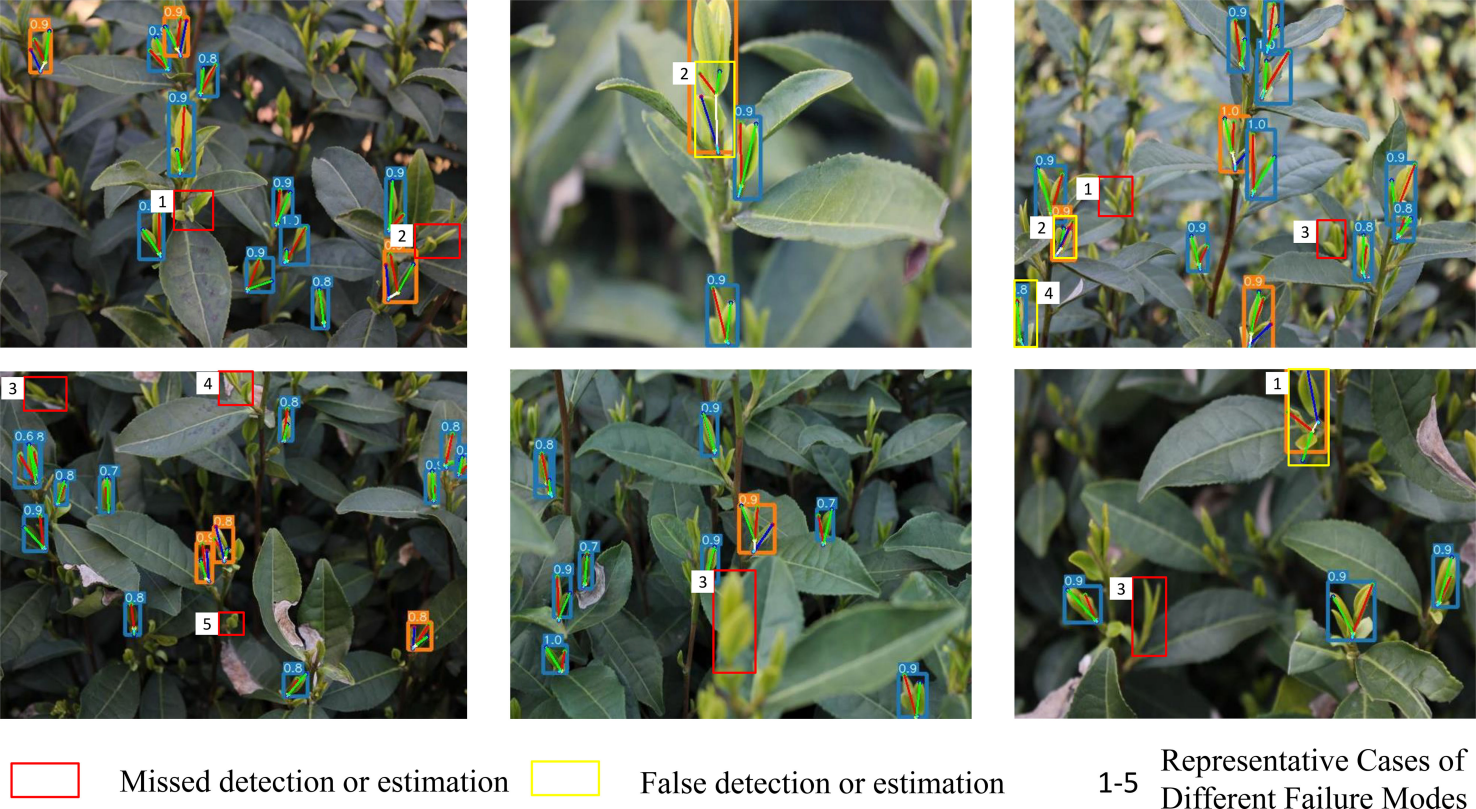
Visualization of failure cases.

To comprehensively evaluate the model’s limitations in practical applications, this section visualizes representative failure cases related to bounding box and key point predictions from the testset (see **Figure 17**) and conducts a root-cause analysis. All samples are derived from real-world tea plantation data and are categorized into five primary failure modes.The serial number of each failure mode corresponds to that in the figure:

(1)Pose Limitations: When buds grow nearly perpendicular to the image plane, their 3D structures collapse into compressed 2D projections, causing spatial ambiguity in keypoint distribution and resulting in pose estimation errors.
(2)Occlusion Interference: Suboptimal shooting angles cause leaves to fully occlude buds, preventing the model from perceiving morphological features and leading to missed detections or erroneous keypoint predictions.
(3)Imaging Distance and Position Constraints: Targets too close or far from the camera result in defocused images, while objects located at image boundaries suffer from partial information loss, degrading detection and keypoint localization accuracy.
(4)Target Truncation: Partially captured buds at image edges lack contextual completeness, causing failures in detection or unreasonable pose outputs.
(5)Excessive Scale Reduction: Extremely small buds produce weak feature responses, making them susceptible to background noise and resulting in missed detections or keypoint offsets.

It is noteworthy that these failure scenarios predominantly occur under extreme or non-ideal acquisition conditions. This analysis not only clarifies the model’s operational boundaries but also provides actionable insights for future improvements.

## 4 Conclusions

The identification of buds and leaves is a crucial part of intelligent tea plucking, but accurately grading and correctly estimating the poses of buds and leaves are also vital for ensuring successful harvesting. Such achievement enables a closed-loop of intelligent tea plucking and allows for grading during harvest, significantly improving efficiency. We successfully developed a tea bud pose estimation and grading detection model based on the improved YOLOv7, and verified the feasibility of deep learning technologies in implementing fine-grained agricultural computer vision tasks under complex field environments.

First, pose estimation was incorporated into the tea bud detection framework, which enabled the model to perform high-precision classification and identification of one-bud-one-leaf and one-bud-two-leaves structures and to simultaneously output the spatial positions of their key points. This achievement provides critical pose and direction information for the picking end-effector, underlies the realization of adaptive flexible picking strategies and promotes the transition of picking operations from ‘recognition and localization’ to the formation of a ‘perceptual decision-making’ closed loop. In terms of methods, ELA and DSConv were introduced to enhance the model attention to tea shoot shape and its ability to capture information. After most CBS modules were replaced with Ghost_conv, the number of parameters was significantly reduced. Meanwhile, the use of EIOU optimized bounding box regression.

Although the model proposed here can achieve accurate detection, grading, and 2D pose estimation of tea buds, it still has some limitations. (1) The data type is singular, but there are countless tea varieties. This experiment focused on two varieties (Longjing 43 and Zhongcha 108), but neglected other tea varieties. (2) Currently, only 2D pose estimation of tea bud images is performed, which can provide references for picking strategies but cannot further support the understanding of 3D spatial poses of tea leaves. In the future, efforts can be made to further optimize model structure, enhance its generalization ability, incorporate depth information, and explore more computer vision technologies suitable for localization of tea picking points.

## Data Availability

The raw data supporting the conclusions of this article will be made available by the authors, without undue reservation.
